# Anthocyanin-Rich Dark Sweet Cherry Phenolics Drive Context-Dependent Modulation of the Nrf2–Keap1–p62 Pathway in Drug-Resistant Triple Negative Breast Cancer Cells: An In Vitro Study

**DOI:** 10.3390/nu18030384

**Published:** 2026-01-24

**Authors:** Ana Nava-Ochoa, Rodrigo San-Cristobal, Susanne U. Mertens-Talcott, Giuliana D. Noratto

**Affiliations:** Department of Food Science and Technology, Texas A&M University, College Station, TX 77843, USA; ana.nava@tamu.edu (A.N.-O.); r.sancristobalblanco@ag.tamu.edu (R.S.-C.);

**Keywords:** triple negative breast cancer, *Prunus avium*, anthocyanins, polyphenols, Nrf2-Keap1-p6w axis, oxidative stress, redox signaling, drug resistance, doxorubicin, in vitro model

## Abstract

Background/Objectives: Triple negative breast cancer (TNBC) is an aggressive subtype treated primarily with chemotherapy, which often leads to drug resistance (DR) and reduced effectiveness. Phytochemicals, including anthocyanins from dark sweet cherry (ACN), have emerged as potential adjuvants to overcome DR, though mechanisms remain unclear. This study examines ACN effects on canonical and non-canonical antioxidant pathways (Nrf2-Keap1 and p62) as a mechanism to overcome DR in 4T1 TNBC cells with acquired DR. Methods: Two conditions were tested: ACN with basal doxorubicin (DOX) as resistance-maintaining conditions and ACN with DOX at IC_50_ to induce oxidative stress (OS). Results: Under resistance-maintaining conditions, ACNs activated the canonical Nrf2-Keap1 pathway at high doses, which can potentially contribute to DR development due to its cellular protection effects. However, at a low dose, ACN did not trigger an antioxidant response linked to GST and GGT enzyme activities and instead impaired autophagy, increasing OS. Under OS, ACN activated the non-canonical antioxidant pathway mediated by p62 while deactivating Nrf2, leading to autophagy-induced cell death and further impairing autophagy at a low dose. Notably, inflammation persisted at both treatment levels without being relieved, keeping stress signaling active. At both conditions, ACN at doses likely attainable under physiological conditions effectively impaired autophagy and elevated OS, resulting in cell death. Conclusions: These results underscore the context-dependent dual function of polyphenols in cancer therapy, demonstrating their potential to enhance cellular sensitivity to chemotherapy and providing guidance for their strategic use as adjuvants in treating TNBC and overcoming DR. However, this study was limited to a single cell line derived from a murine model. Future research should include comparative studies using human TNBC cell lines to validate these findings and better assess their translational relevance.

## 1. Introduction

Triple negative breast cancer (TNBC) is characterized by the absence of three key receptors: human epidermal growth factor receptor 2 (HER2), estrogen receptor (ER), and progesterone receptor (PR). Due to this lack of receptor expression, TNBC cannot be treated with endocrine therapy or receptor-targeted drug therapies, which are commonly used in other forms of breast cancer (BC) [1]. TNBC is among the most aggressive types of BC and is notably associated with heightened drug resistance (DR), a common effect of conventional cancer treatments. This resistance contributes to the cancer’s increased aggressiveness and invasiveness, often leading to metastasis. DR in cancer cells arises from genetic or epigenetic mutations, enabling them to evade the effects of therapies targeting specific molecular markers [2]. One mechanism by which this occurs is the upregulation of antioxidant defenses mediated by the Nrf2–Keap1–p62 axis. This pathway enhances cellular capacity to escape oxidative stress (OS), allowing cancer cells to withstand oxidative stress induced by chemotherapeutic agents. As a result, drug efficacy is significantly reduced, making this axis clinically relevant as a potential therapeutic target for overcoming resistance and improving treatment outcomes [3].

The antioxidant pathway mainly involves the nuclear factor erythroid 2–related factor 2 (Nrf2) transcription factor. Under basal conditions, Nrf2 is bound to Kelch-like ECH-associated protein 1 (Keap1), which promotes Nrf2’s ubiquitination and subsequent degradation. When the binding of Keap1 and Nrf2 is disrupted, the antioxidant defense mechanism is activated through either the canonical or non-canonical antioxidant pathway [4].

The canonical Nrf2 pathway is activated by OS–mediated modification of Keap1, allowing Nrf2 nuclear translocation and binding to antioxidant response elements (AREs) to induce cytoprotective genes. These include phase II detoxifying enzymes such as NADPH quinone oxidoreductase 1 (NQO1), heme oxygenase 1 (HO-1), glutathione S-transferase (GST), and γ-glutamyl transferase (GGT), which protect cells against oxidative damage and xenobiotics [5]. Increased glutathione (GSH) production is a central component of this response and contributes to DR in cancer cells by limiting OS-induced damage [6]. GST and GGT are frequently overexpressed in DR cells and promote GSH-dependent drug detoxification and elimination, thereby reducing chemotherapeutic efficacy [7,8,9]. NF-κB, an OS-responsive inflammatory transcription factor, has also been linked to the regulation of antioxidant genes such as NQO1 and HO-1 [10].

The non-canonical pathway involves OS-induced autophagy, which plays a context-dependent role in cancer by promoting either cell survival or autophagic cell death [11,12]. The autophagy adaptor p62 can sequester Keap1, sustaining Nrf2 activation and contributing to DR through prolonged antioxidant signaling [4]. However, excessive p62 accumulation may also promote stress-induced autophagic cell death [13]. Accordingly, inhibition of Nrf2 has been shown to restore drug sensitivity in DR cancer cells by enhancing OS-mediated cytotoxicity [3].

Polyphenols, naturally occurring compounds found in plant-based foods, have demonstrated health benefits including anti-inflammatory, antioxidant, and anticancer properties [8,9]. Their use in cancer therapy has been proposed as a strategy to overcome DR and metastasis, particularly when combined with chemotherapeutic agents. This is due to their reported synergistic anticancer effects, which include targeting the cell cycle, modulating signal transduction pathways, upregulating tumor suppressing genes, downregulating oncogenes, and inducing apoptosis, mechanisms that demonstrate enhanced efficacy in combination [14]. Studies have shown that polyphenols, both alone and in combination with chemotherapy, can effectively counteract DR [15,16,17]. Dark sweet cherries (DSC) are a rich source of polyphenols. Notably, the anthocyanin-rich fraction (ACN) of DSC has shown to induce apoptosis in cancer cells, modulate cell signaling pathways in vitro, and suppress tumor growth in vivo [18,19,20,21]. Moreover, research has demonstrated that ACN from DSC synergize with doxorubicin (DOX) by targeting phase I metabolizing enzymes and phase III drug efflux transporters [22]. Polyphenols have been evaluated on their effect on TNBC for their ability to modulate signaling pathways and transcription factors, including Nrf2. Evidence indicates that their regulatory effect on Nrf2 is context-dependent, exerting either activation or suppression based on cellular conditions. This modulation is particularly relevant when polyphenols are combined with chemotherapeutic agents, as Nrf2-targeted regulation has been associated with enhanced drug efficacy and reduced tumor progression, highlighting their potential as adjuvant therapeutic strategies [1,23]. These results show potential to evaluate ACN from DSC within the context of DR.

The objective of this study was to explore the effects of ACN on 4T1 breast cancer DR cells, which resemble the characteristics of stage IV human breast cancer with acquired resistance to drugs. Specifically, we aimed to elucidate the Nrf2-linked antioxidant mechanisms modulated by ACN in 4T1 BC cells with developed DR to DOX under resistance-maintaining and OS conditions to evaluate its effect in sustaining DR.

## 2. Materials and Methods

### 2.1. Chemicals, Antibodies, and Reagents

Solvents used to prepare the ACN extract as well as Bovine Serum Albumin (BSA) were purchased from Fisher Chemical (Pittsburgh, PA, USA). Doxorubicin HCl was purchased from Enzo Life Sciences (Farmingdale, NY, USA). Cyanidin 3-O-Rutinoside was purchased from Alladin Scientific (Riverside, CA, USA). 1-Chloro-2,4-dinitrobenzene (CDNB), Glutathione (GSH), and 2,7-Dichlorofluerescin diacetate (H2DCFDA) were purchased from Sigma Aldrich (Saint Louis, MO, USA). Fetal bovine serum (FBS) was purchased from Gibco, Invitrogen Corp. (Grand Island, NY, USA). Penicillin–streptomycin antibiotic mix (P/S), Halt™ Protease Inhibitor Cocktail (100×), and Invitrogen NuPAGE LDS Sample Buffer were purchased from ThermoFisher Scientific (Grand Island, NY, USA). The xTractor™ buffer was purchased from Takara Bio Company (Mountain View, CA, USA). Western Blotting Luminol Reagent was purchased from Santa Cruz Biotechnology (Dallas, TX, USA). Protein dye reagent for Bradford assay, precision plus protein dual color protein ladder, Supermix iScript™, and SsoAdvanced™ Universal SYBR^®^ Green Supermix were purchased from BioRad Laboratories (Hercules, CA, USA). Antibodies for p62/KEAP1/NRF2 pathway (SQSTM1/p62, phospho-SQSTM1/p62, LC3I/II, KEAP1, NRF2, HO-1, NQO1), GAPDH, NF-κB, phospho-NF-κB, and the secondary anti-rabbit IgG, HRP-linked antibody were purchased from Cell Signaling Technology Inc. (Danvers, MA, USA).

### 2.2. Extraction of Anthocyanin-Rich Fraction (ACN) from DSC Concentrated Juice

Concentrated DSC juice was kindly supplied by FruitSmart Inc. (Grandview, WA, USA). Its phenolic profile was previously reported [24]. Fractionation of phenolic compounds and extraction of a phenolic fraction rich in anthocyanins (ACN) was performed as previously reported [20]. Quantification of anthocyanins in the ACN extract was performed by the pH-differential spectrophotometric method [25] as cyanidin-3-O-rutinoside equivalents (C3R Eq.), which is the most abundant anthocyanin in the ACN rich fraction. C3R accounts for 79% of the total anthocyanins in concentrated DSC juice, followed by cyanidin 3-O-glucoside and peonidin-3-O-rutinoside, which account for 9% and 8% of total anthocyanins, respectively [24]. ACN extraction and anthocyanins quantification were performed before each experiment to ensure the standardization of the ACN extract.

### 2.3. Cell Line

4T1 cells derived from the mouse mammary gland were purchased from ATCC (Manassas, VA, USA). Cells were grown in RPMI-1640 culture medium purchased from ATCC (catalog #30-2001) supplemented with 10% (*v*/*v*) FBS and 1% (*v*/*v*) P/S. Cells were maintained at 37 °C with a humidified 5% CO_2_ atmosphere, as recommended by ATCC.

### 2.4. Drug Resistance (DR) Development

The 4T1 cells were cultured in media supplemented with DOX as previously reported [26] with slight modifications. Briefly, cells maintained in culture media supplemented with 20 nM DOX were passed, upon reaching confluence, into a new media supplemented with increased DOX dose by 10 nM up to 100 nM. DR cells were kept in media containing 100 nM DOX throughout the study. To evaluate DR development, cell viability was assessed as detailed in Section 2.5.

### 2.5. Cell Viability

Cell viability was assessed using the Resazurin *in vitro* toxicology assay kit (Sigma-Aldrich, St Louis, MO, USA) following the manufacturer’s protocol. Briefly, cells seeded in 96-well plates were allowed to reach 75% confluence, followed by treatment with ACN (0–200 μg C3R Eq./mL) and/or DOX (0–20 μg/mL) for 48 h. A pure C3R standard (0–200 μg/mL) was used to compare cell viability results with ACN treatments. Relative fluorescence units (RFU) were measured at 560 nm and 590 nm excitation and emission wavelengths, respectively, using the ClarioStar plate reader (BMG Labtech Inc, Durham, NC, USA). Cell viability was represented as % of control from *n* ≥ 3 repetitions, calculated as percentage of dimethyl sulfoxide (DMSO)-treated control cells. The dose of ACN, DOX, and C3R needed to inhibit cell viability by 50% (IC_50_) was calculated from at least three independent determinations. Untreated controls (control) contained DOX at its resistance-maintaining dose (100 nM) and up to 0.2% *v*/*v* DMSO used as a solvent of ACN and DOX.

### 2.6. Gene Expression

DR cells seeded in 6-well plates were allowed to reach ~85–90% confluence, followed by treatment with ACN at different ratios of its IC_50_: 0.5 × IC_50_, 1 × IC_50_, and 1.5 × IC_50_ in the presence of DOX in culture medium supplemented with 2.5% FBS. DOX treatments consisted of a maintenance dose (100 nM) to maintain resistance or DOX IC_50_ to induce OS. The incubation period was selected based on the observed maximum modulation of the target genes of interest within a 2–6 h timeframe. The mRNA extraction from cells was performed using the Zymo Quick-RNA™ Microprep Kit (Zymo Research, Irvine, CA, USA) following the manufacturer’s protocol. The reverse transcription Supermix iScript™ was used for cDNA synthesis, followed by real-time polymerase chain reaction (RT-PCR) amplification using SsoAdvanced™ Universal SYBR^®^ Green Supermix. Mouse ribosomal protein L19 (RPL-19) and β-actin were tested as housekeeping genes, and RPL-19 was chosen for its minimal inter-sample variability. Relative mRNA levels were calculated employing the comparative CT method as described [27]. Gene’s primer sequences are presented in Table 1.

### 2.7. Protein Expression

DR cells were seeded onto 10 cm culture plates and allowed to reach ~85% confluence. Cells were starved overnight in 2.5% FBS medium followed by treatment with ACN at different ratios of its IC_50_: 0.5 × IC_50_, 1 × IC_50,_ and 1.5 × IC_50_ in presence of DOX at resistance-maintaining dose (100 nM) or DOX IC_50_ in culture medium with FBS-free medium for 24 h. Cells lysates were obtained using xTractor buffer and protease inhibitor cocktail following manufacturer’s protocol accompanied by mechanical cell disruption (series of freezing, thawing, and sonication for 30 s), followed by centrifugation at 10,000× *g* and 4 °C for 10 min. Supernatant was subjected to Western blot analysis as reported [24] using 35 μg of protein quantified against a BSA standard curve with the Bradford protein assay. Protein bands were visualized using Western blotting luminol reagent after 1–5 min of reaction. Band intensities were quantified using ImageJ Software (ImageJ.JS version 1, https://imagej.net/ij/).

### 2.8. GST Enzymatic Activity

GST activity was assessed by quantifying the production of CDNB by GSH as previously reported [28] with slight modifications. Briefly, DR cells were treated with ACN at different ratios of its IC_50_: 0.5 × IC_50_, 1 × IC_50_, and 1.5 × IC_50_ in the presence of DOX at resistance-maintaining dose (100 nM) or DOX IC_50_ in culture medium with FBS-free medium for 24 h in a serum-free medium. After treatment, cells were washed with PBS and collected with ice-cold PBS containing 0.1% triton-X100. Cells were ultrasonicated in cold to lyse cell membrane and centrifuged at 4000× *g* for 5 min at 4 °C. Supernatant was used to measure GST activity in 96 well plates. Activity assay consisted of adding 10 µL of cell lysate to each well in the presence of 170 µL of 100 mM potassium phosphate buffer pH = 6.5 and 10 µL of 100 mM GSH in H2O (pH = 7.0). To start the reaction, 10 µL of CDNB at 40 mM in EtOH was added to each well. Activity was assessed by reading absorbance at 340 nm every 90 s for 100 min using the ClarioStar plate reader. GST activity was assessed using the absorbance data on the time points at which the reaction was exponential. CDNB’s molar extinction coefficient (ε) of 0.0096 µM^−1^cm^−1^ and well path length (l) of 0.524 cm was used in the following formula:

(1) $$GST\;activity\;(U)=\left(\frac{\left(\Delta Abs\right)}{\left(\Delta time\right)}\right)\times \left(\frac{Total\;reaction\;volume}{\varepsilon \;\times Sample\;volume\times l}\right)\times DF$$

U accounts for unit of enzyme that conjugates 1 µmol of CDNB with reduced GSH per min (µmol/min/mL). Protein content in cell lysates was quantified by Bradford protein assay to normalize GST activity to U/µg of protein.

### 2.9. GGT Enzyme Activity

GGT activity was quantified by measuring the production of *p*-nitroaniline (PNA) by cell lysates using the γ Glutamyl transferase activity assay kit by Cayman Chemical (Ann Arbor, MI, USA) following manufacturer’s protocol. Briefly, GGT activity was measured by adding 90 µL of GGT reaction buffer and 10 µL of cell lysate prepared as detailed in Section 2.8, to a well in a 96-well plate. A reaction background was included by adding only reaction buffer to the wells with no cell lysate. Absorbance was read at 418 nm every minute at 37 °C using the ClarioStar plate reader. GGT activity was assessed using the absorbance data on the time points at which the reaction was exponential. PNA’s molar absorption coefficient (ε) of 0.0088 µM^−1^cm^−1^ and well path length (l) of 0.28 cm were used to quantify GGT activity as follows:

(2) $$GGT\;activity\;(U)=\left(\frac{\left(\Delta Abs\right)}{\left(\Delta time\right)}\right)\times \left(\frac{Total\;reaction\;volume}{\varepsilon \times Sample\;volume\times l}\right)\times DF$$

U accounts for the unit of enzyme that conjugates 1 µmol of PNA per min (µmol/min/mL). Protein content in cell lysates was quantified by Bradford protein assay to normalize GGT activity to U/µg of protein.

### 2.10. Reactive Oxygen Species (ROS) Generation

Generation of ROS was quantified as reported [29] with slight modifications. Briefly, cells seeded in 96-well plates were incubated until 100% confluence. Cells were pretreated with ACN at different ratios of its IC_50_: 0.5 × IC_50_, 1 × IC_50_, and 1.5 × IC_50_ for 2 h followed by addition of DOX treatment at resistance-maintaining dose and DOX IC_50_ for 2 h. Cells were washed twice with PBS followed by 30 min incubation with 10 µM CM-H2DCFDA under dark at 37 °C. Fluorescence was read using ClarioStar plate reader at 480 nm excitation and 525 nm emission. Results represent relative fluorescence units (RFU) but are reported as fold of control.

### 2.11. Statistical Analysis

To ensure consistency and comparability, the number of biological replicates was standardized across all experimental analyses. Unless otherwise specified, each experiment was conducted using *n* ≥ 3 independent biological replicates. Quantitative data is presented as mean values with the respective standard deviation (SD) or standard error of the mean (SEM). Significant difference between treatments was determined by one-way analysis of variance (ANOVA) followed by Tukey’s multiple comparisons test using GraphPad Prism 10 (Systat Software, Inc., San Diego, CA, USA). Results are expressed as fold of control which refers to cells exposed to DOX resistance-maintaining dose.

The correlation heatmap was generated using GraphPad Prism by performing a two-tailed Pearson correlation analysis with a 95% confidence interval. Only correlations with *p*-values less than 0.05 were included in the heatmap.

## 3. Results

### 3.1. Development of a Drug-Resistant Cell Line Alters Viability Response to ACN and DOX

Cell viability results confirmed the acquisition of resistance to DOX in 4T1 parental cells. Results showed that cell viability of cells resistant to DOX (4T1-DR) was significantly increased compared to parental 4T1 cells (Table 2).

**Figure 1:**
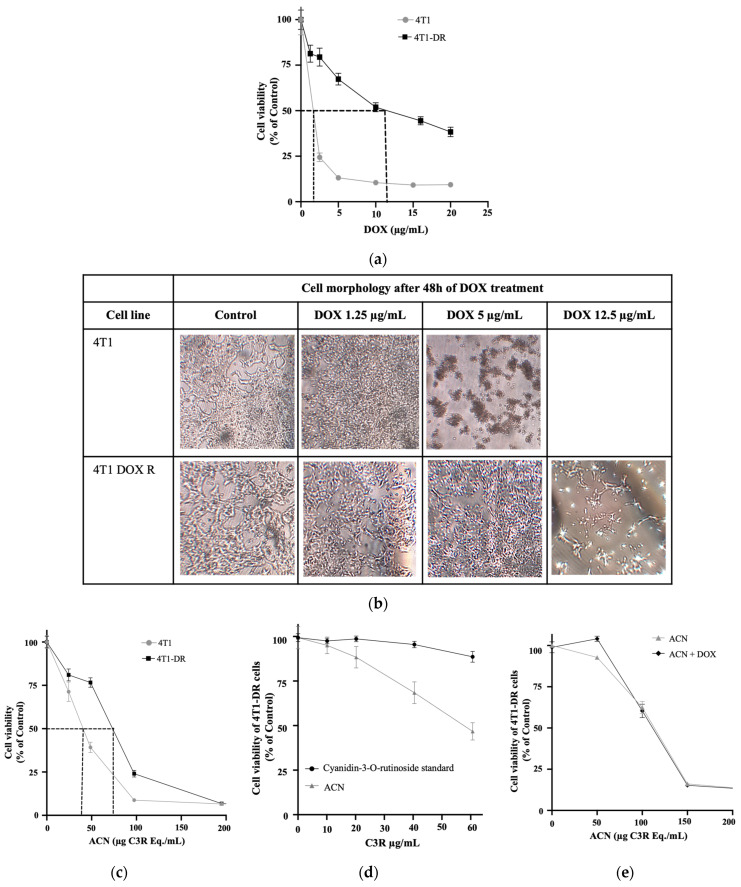
Prolonged exposure to DOX resulted in the development of a drug-resistant cell line, which exhibited a distinct viability response to both ACN and DOX compared to the parental cell line. (**a**) Cell viability of the 4T1 cell line and drug-resistant 4T1-DR cell line following DOX treatment (0–20 µg/mL) for 48 h. The DOX IC_50_ for 4T1 cells was ~1.78 µg/mL, whereas for 4T1-DR cells, it was ~10.75 µg/mL. (**b**) Representative morphological changes in parental 4T1 cells (top panel) versus 4T1-DR cells (lower panel) after exposure to increasing concentrations of DOX. (**c**) Cell viability of 4T1 and 4T1-DR cell lines treated with ACN (0–200 µg C3R Eq./mL) for 48 h. The 4T1-DR cells achieved IC_50_ at a higher dose (73.48 ± 0.08 µg C3R Eq./mL) compared to parental 4T1 cells (40.46 ± 0.26 µg C3R Eq./mL). (**d**) Cell viability of 4T1-DR cells treated with ACN (0–60 µg C3R Eq./mL) or pure C3R (0–60 µg/mL). Treatment with ACN resulted in cell viability of 46.73 ± 4.84% of untreated control, while C3R showed 88.57 ± 8.65% at the highest tested dose (60 µg/mL). (**e**) Cell viability of 4T1-DR cells treated with ACN (0–200 µg C3R Eq./mL) in the presence and absence of the DOX resistance-maintaining dose for 48 h. No significant difference in cell viability was observed between treatments with or without DOX resistance-maintaining dose. Cell viability (% of DMSO control) was evaluated utilizing the resazurin assay, as described in Section 2. Values presented represent mean (*n* ≥ 3) ± SD. Curves reflect results of more than one independent experiment.

As illustrated in Figure 1a, 4T1-DR cells treated with DOX (0–20 µg/mL or 0–37 nM) showed an IC_50_ of 10.66 ± 2.91 µg/mL, while in the 4T1 parental cells, the IC_50_ was 1.78 ± 0.22 µg/mL. The morphological changes in both the 4T1 and 4T1-DR were visually assessed to confirm DOX resistance (Figure 1b).

The 4T1-DR cells also developed resistance to ACN as shown in Figure 1c, Table 3, where cell viability was inhibited to 76.61% when treated with 50 µg C3R Eq./mL, while in the parental 4T1 cells cell viability was inhibited to 39.21%. Moreover, it was confirmed that the combination of anthocyanins present in the ACN extract, mainly C3R (80%), Cyanidin-3-O-glucoside (C3G, 10%), Peonidin-3-O-rutinoside, and others unidentified (10%) [24], was more effective in suppressing the viability of 4T1-DR cells.

As shown in Figure 1d, Table 4, cells treated with 60 µg C3R eq./mL had their cell viability inhibited by 53.27%, while in cells treated with the C3R standard at 60 µg/mL, their cell viability was inhibited by only 11.43%. This enhanced effect may be attributed to the synergistic interactions among polyphenols present in the ACN extract, which have been shown to amplify anticancer activity beyond the effects of individual compounds [14].

It was noted that 4T1-DR cells became more resistant to ACN after each passage. Therefore, cell viability tests were made before each experiment and the respective IC_50_ for each passage was used for each experiment.

Lastly, to confirm that the DOX resistance-maintaining dose (100 nM) did not have a significant cytotoxic effect in 4T1-DR cells, the effect of ACN (0–150 µg/mL) on cell viability was assessed in the presence and absence of DOX at 100 nM. Results showed no significant difference between cell viability dose response in presence of DOX (IC_50_ = 114.82 ± 5.03 µg C3R Eq./mL) and absence of DOX (IC_50_ = 113.14 ± 4.98 µg C3R Eq./mL) (Figure 1e, Table 5).

### 3.2. Low-Dose ACN Minimally Activates Nrf2–Keap1 While Impairing Autophagy Under Resistance-Maintaining Conditions

**Figure 2:**
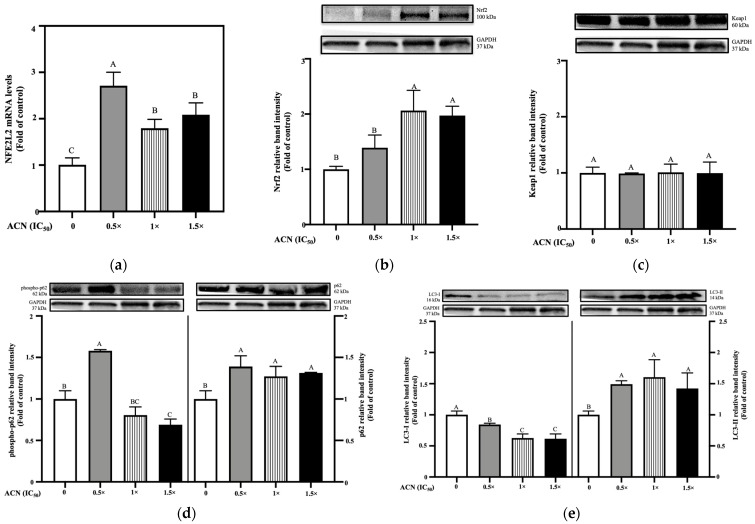
ACN at highest doses activates the Nrf2 pathway under resistance-maintaining conditions by upregulating Nrf2 expression independently of autophagy, as indicated by the absence of phosphorylated p62 (phospho-p62) interaction with Keap1, whereas ACN at 0.5 × IC_50_ does not trigger Nrf2 pathway activation and impairs autophagy. (**a**) Relative mRNA levels of Nfe2l2. (**b**) Protein expression levels of Nrf2. (**c**) Protein expression levels of Keap1. (**d**) Protein expression levels of phospho-p62 (**left** panel) and p62 (**right** panel). (**e**) Ratio of LC3-II/LC3-I protein expression. DOX concentration was maintained at 100 nM for all treatments. Cells were treated with ACN (0.5 × IC_50_, 1 × IC_50_, and 1.5 × IC_50_) for 6 h for mRNA expression and 24 h for protein expression. All values are expressed as fold-change relative to DMSO control and presented as mean *(n* ≥ 3) ± SD. Protein expression results show each protein’s relative band intensity on the top panel and loading controls using GAPDH in the lower panel. Significant differences between treatments were determined using ANOVA and are indicated by different letters. mRNA and protein expression analyses were performed as described in Section 2.

Results showed that under resistance-maintaining conditions, which might resemble homeostatic conditions on DR cells, Nfe2l2 gene expression (which encodes the Nrf2 transcription factor) was upregulated in 4T1-DR treated with ACN (0.5 × IC_50_, 1 × IC_50_, and 1.5 × IC_50_) up to 2.71-, 1.79-, and 2.08-fold of control, respectively (Figure 2a).

Consistent with mRNA levels, protein levels of Nrf2 increased in 4T1-DR cells treated with ACN (0.5 × IC_50_, 1 × IC_50_, and 1.5 × IC_50_) up to 1.40-, 2.06-, and 1.96- fold of control, respectively (Figure 2b). However, ACN at 0.5 × IC_50_ did not reach significant difference against control. ACN treatments did not modulate Keap1 protein levels compared to control at any level (Figure 2c).

To assess whether Nrf2 activation is mediated by p62 competing with Keap1, both total p62 and its phosphorylated form (phospho-p62) were measured. Phosphorylation of p62 significantly enhances its binding affinity for Keap1, thereby promoting sequestration of Keap1 into autophagosomes and facilitating Nrf2 pathway activation [30]. High phospho-p62 along with accumulation of total p62, points to impaired autophagic flux. This impairment is characterized by a buildup of damaged organelles and increased endoplasmic reticulum stress, ultimately predisposing cells to death [31]. Only ACN at 0.5 × IC_50_ showed higher expression of phospho-p62 than control and other ACN doses at 1.58-fold of control (Figure 2d, left panel). At this ACN dose, p62 at its total form showed higher band intensity levels at 1.39-fold of control, implying impaired autophagy (Figure 2d, right panel). Both ACN 1 × IC_50_ and 1.5 × IC_50_ did not show significant difference against control in phospho-p62, while total p62 showed higher band intensity levels in both ACN 1 × IC_50_ and 1.5 × IC_50_ to 1.27- and 1.31-fold of control, respectively (Figure 2d, right panel). Since Keap1 binding depends on its interaction with phospho-p62, these results suggest that Nrf2 activation occurred independently of p62-mediated Keap1 sequestration. To confirm the role of p62 on autophagy induction, the conversion of LC3-I (cytosolic form) to LC3-II (lipidated form) protein expression was evaluated. Treatment with ACN (0.5 × IC_50_, 1 × IC_50_ and 1.5 × IC_50_) resulted in decreased LC3-I protein expression to 0.84-, 0.62-, and 0.61-fold of control, respectively (Figure 2e, left panel). Consequently, LC3-II band intensity was increased by ACN (0.5 × IC_50_, 1 × IC_50_ and 1.5 × IC_50_) to 1.49-, 1.60, and 1.42-fold of control, respectively (Figure 2e, right panel). The stable presence of p62 indicates autophagy machinery is active with higher LC3-I to LC3-II conversion, supporting the activation of autophagy. However, at lower dose, high presence of phospho-p62 and p62 indicate impaired autophagy.

**Figure 3:**
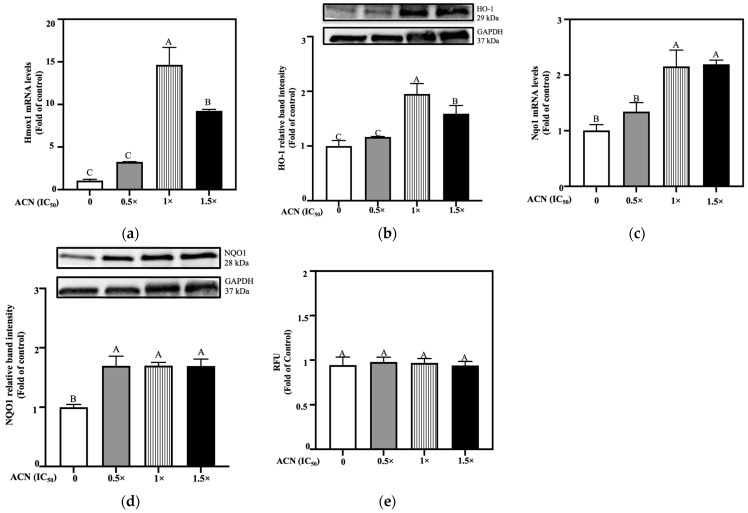
ACN induced the expression of Heme oxygenase-1 (Hmox1) and NADPH quinone dehydrogenase 1 (NQO1) in 4T1-DR cells via ARE-mediated regulation under resistance-maintaining conditions. (**a**) Relative mRNA levels of Hmox1. (**b**) Protein expression levels of HO-1, encoded by Hmox1. (**c**) Relative mRNA levels of Nqo1. (**d**) Protein expression levels of NQO1, encoded by Nqo1. (**e**) Levels of reactive oxygen species (ROS). DOX concentration was maintained at 100 nM for all treatments. Cells were treated with ACN (0.5 × IC_50_, 1 × IC_50_, and 1.5 × IC_50_) for 6 h for mRNA expression, 24 h for protein expression, and 4 h for ROS quantification. All values are expressed as fold-change relative to DMSO control and presented as mean (*n* ≥ 3) ± SD. Protein expression results show each protein’s relative band intensity on the top panel and loading controls using GAPDH in lower panel. Significant differences between treatments were determined using ANOVA and are indicated by different letters. mRNA and protein expression analyses were performed as described in Section 2.

AREs are DNA sequences that regulate the expression of antioxidant-related genes, and Nrf2 is the transcription factor that activates them. Therefore, their upregulation following Nrf2 upregulation and translocation to the nucleus indicates activation of the canonical Nrf2 signaling pathway, leading to the expression of Nrf2 target genes including NQO1, HO-1, GST, and GGT. The Hmox1 gene encodes the HO-1 protein, which plays a key role in neutralizing ROS. In this study, Hmox1 mRNA levels were upregulated by ACN treatment (0.5 × IC_50_, 1 × IC_50_, and 1.5 × IC_50_), up to 3.26-. 14.65, and 9.23-fold of control, respectively (Figure 3a). Consistently, HO-1 protein levels were upregulated to 1.16-, 1.95-, and 1.59-fold of control for ACN at 0.5 × IC_50_, 1 × IC_50_, and 1.5 × IC_50_, respectively (Figure 3b). No significant differences against control were detected for this marker at mRNA and protein levels at for ACN at 0.5 × IC_50_.

Expression of Nqo1 gene, which encodes the NQO1 protein, contributes to antioxidant defense by preventing redox cycling and thereby reducing ROS levels. Results showed that ACN treatment (1 × IC_50_, and 1.5 × IC_50_) upregulated NQO1 mRNA levels significantly up to 2.15- and 2.19-fold of control, respectively (Figure 3c). NQO1 protein levels showed significant increase against control across all ACN treatments, reaching 1.74-fold of control at ACN 0.5 × IC_50_ and 1 × IC_50_, and 1.73-fold of control at 1.5 × IC_50_ (Figure 3d). No significant alterations in ROS levels were detected following ACN exposure, suggesting a possible adaptive state in which ROS is not cleared by these markers but still showing that ACN does not induce OS (Figure 3e).

Expression levels of genes regulated by AREs provide important information about the activation of cellular antioxidant pathways; however, the enzymes governed by this pathway are crucial for the detoxification of drugs and xenobiotics. Therefore, it is essential to assess not only gene and protein expression, but also the activity of the main enzymes in this pathway, as enzyme activity directly reflects functional detoxification capacity. Both GST and GGT enzymes play key roles, with GGT expression linked to DR and its activity proposed as a marker for carcinogenesis due to its role in cancer cell promotion and invasiveness. Likewise, GST is central to drug detoxification and regulation of OS, making enzyme activity assays a vital indicator of pathway function [32].

**Figure 4:**
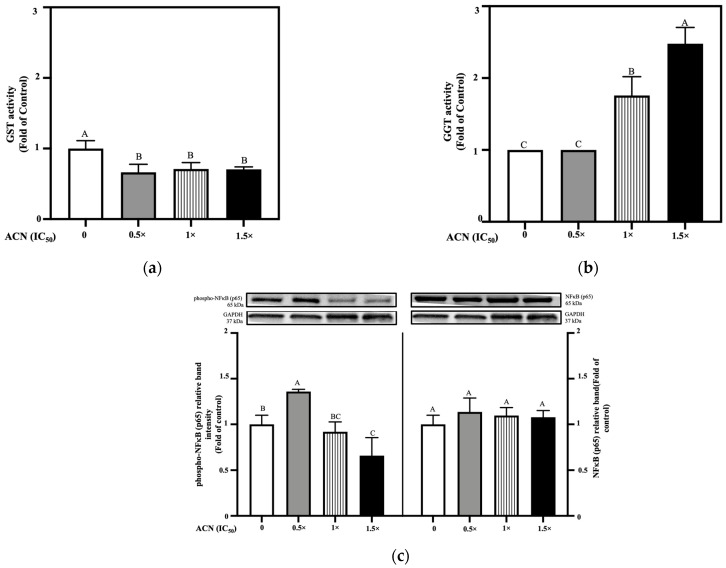
ACN-modulated key antioxidant enzyme activities and inflammatory signaling in 4T1-DR cells under resistance-maintaining conditions. (**a**) GST enzyme, (**b**) GGT enzyme activity, and (**c**) phospho-NF-κB and NF-κB p65 protein expression. DOX concentration was maintained at 100 nM for all treatments. Cells were treated with ACN (0.5 × IC_50_, 1 × IC_50_, and 1.5 × IC_50_) for 24 h for both enzymatic activity and protein expression. All values are expressed as fold-change relative to DMSO control and presented as mean (*n* ≥ 3) ± SD. Protein expression results show each protein’s relative band intensity on the top panel and loading controls using GAPDH in lower panel. Significant differences between treatments were determined using ANOVA and are indicated by different letters. Enzymatic activity and protein expression analyses were performed as described in Section 2.

Results showed that GST activity was suppressed when cells were treated with ACN at 0.5 × IC_50_, 1 × IC_50_ and 1.5 × IC_50_ down to 0.63-, 0.71-, and 0.70-fold of control, respectively (Figure 4a).

On the contrary, GGT activity was induced but only by ACN at 1.0 × IC_50_ and 1.5 × IC_50_, up to 1.75- and 2.47-fold of control, respectively (Figure 4b). Lastly, under these conditions, treatment with ACN at 0.5 × IC_50_ showed an increase in phospho-NF-κB (p65) levels to 1.36-fold of control while ACN at 1.0 × IC_50_ and 1.5 × IC_50_ suppressed these levels to 0.91- and 0.65-fold of control, respectively (Figure 4c, left panel), implying inflammation downregulation. NF-κB (p65) protein levels remained unchanged at all treatment levels (Figure 4c, right panel).

### 3.3. ACN Activates Non-Canonical Nrf2–Keap1 Pathway Under Oxidative Stress to Promote Autophagy

**Figure 5:**
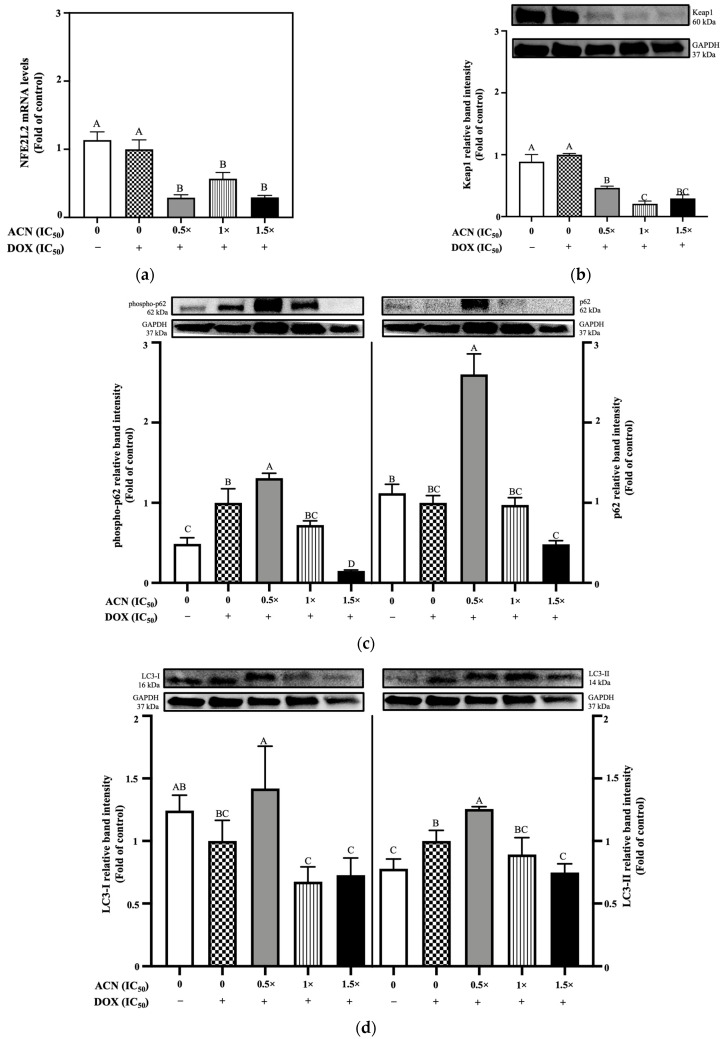
ACN disrupts the Nrf2 pathway under oxidative stress conditions by downregulating Nrf2 and Keap1 markers, while concurrently inducing autophagy through upregulation of *p*-62 and LC3-II in 4T1-DR cells. (**a**) Relative mRNA levels of Nfe2l2. (**b**) Protein expression levels of Keap1. (**c**) Protein expression levels of phosphorylated-p62 (**left**) and basal p62 (**right**). (**d**) Protein expression levels of LC3-I (**left**) and LC3-II (**right**). For all treatments, 4T1-DR cells were incubated with ACN at various IC_50_ ratios in the presence of DOX at 10.75 µg/mL. Treatments for mRNA quantification were maintained for 6 h and for protein expression analysis for 24 h. All results are expressed as fold-change relative to OS control and presented as mean (*n* ≥ 3) ± SD. Different letters indicate statistically significant differences between treatments, as determined by ANOVA. Symbols (− and +) indicate absence or presence of DOX IC_50_, respectively. mRNA and protein expression levels were quantified as described in Section 2.

Results showed that Nfe2l2 mRNA remained at similar levels between 4T1-DR and 4T1-DR under OS cells. Treatment with ACN at 0.5 × IC_50_, 1 × IC_50_, and 1.5 × IC_50_ resulted in significant downregulation of Nfe2l2 mRNA levels compared to the oxidative stress control, reducing expression to 0.29, 0.56, and 0.30-fold, respectively (Figure 5a).

Nrf2 protein expression was assessed by immunoblotting; however, no detectable bands were observed. Keap1 protein was also assessed. Its downregulation is driven by autophagy through its interaction with p62, a process that becomes more rapid when Keap1 is modified under OS conditions, making it a favored substrate for autophagic clearance [33]. Under OS, ACN at 0.5 × IC_50_, 1 × IC_50_, and 1 × IC_50_, promoted a dose-dependent downregulation to 0.46-, 0.20-, and 0.29-fold of OS-control, respectively (Figure 5b). These results were accompanied by changes in the phospho-p62 and p62 levels, showing significantly higher levels of phospho-p62 and the basal p62 (Figure 5c), indicating impaired autophagy by treatment with ACN at 0.5 × IC_50_ dose. However, higher ACN doses seemed to reactivate autophagy flux and clearance, indicated by the decrease in p62 at its basal form. Likewise, LC3-I and LC3-II protein levels were also affected by ACN where treatment with 0.5 × IC_50_ showed a presence of both LC3-I and LC3-II at levels higher than the control. However, treatment with ACN at 1 × IC_50_ and 1.5 × IC_50_ showed a decrease on LC3-I levels compared to OS-control (Figure 5d). These results strongly suggest ACN treatment at a lower dose promotes impaired autophagic flux while higher doses induced autophagic activity. Active autophagic activity and flux, paired with increased phospho-p62 at higher ACN treatments, suggests Keap1 sequestration by phospho-p62 due to its higher affinity at its phosphorylated form, explaining Keap1’s downregulation under OS conditions.

It has been established that p62 has a dual regulatory relationship with Nrf2. First, Nrf2 nuclear translocation and activation of AREs directly induce p62 expression, forming a positive feedback loop in which elevated p62 can further potentiate Nrf2 signaling. Second, phospho-p62 preferentially interacts with Keap1 and competes with Nrf2 for binding, leading to Keap1 sequestration and facilitating the release and nuclear translocation of Nrf2, which enables activation of the Keap1-Nrf2 antioxidant pathway and its target genes. Because of this, the undetected presence of Nrf2 at protein levels might be contributing to low p62 levels, suggesting no regeneration after degradation by autophagy.

**Figure 6:**
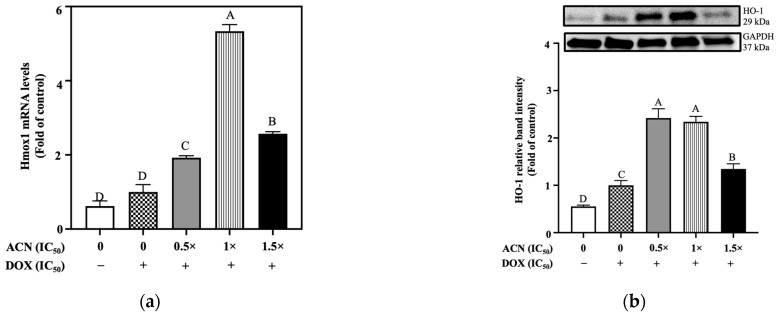

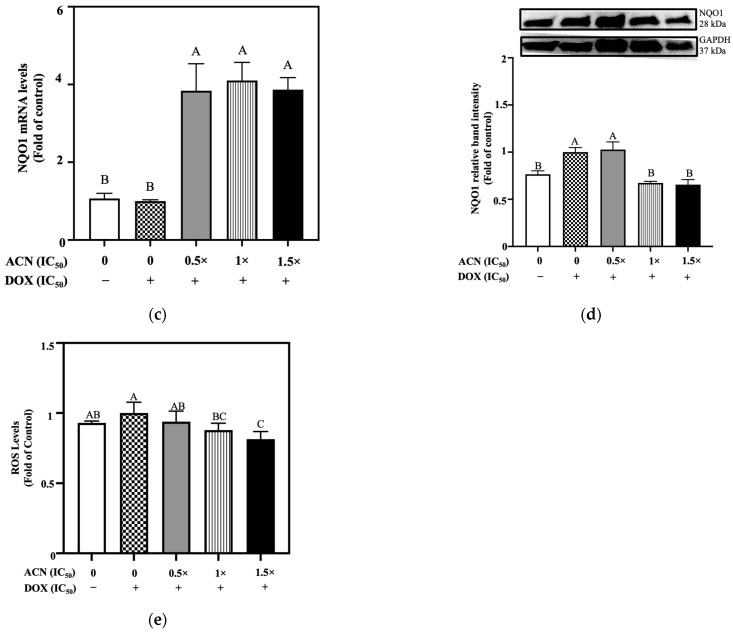
ACN activated antioxidant response elements under oxidative stress by upregulating markers for Heme oxygenase-1 (Hmox1) and NADPH quinone dehydrogenase 1 (NQO1) in 4T1-DR cells. (**a**) Relative mRNA levels of Hmox1 after ACN treatment at various IC_50_ ratios in the presence of DOX at IC_50_ dose (10.75 µg/mL). (**b**) Protein expression levels of HO-1, encoded by Hmox1. (**c**) Relative mRNA levels of Nqo1. (**d**) Protein expression levels of NQO1, encoded by Nqo1. (**e**) Levels of reactive oxygen species (ROS) following ACN treatment at different doses in the presence of DOX. For all treatments, 4T1-DR cells were incubated with ACN at various IC_50_ ratios in the presence of DOX at 10.75 µg/mL. Treatments for mRNA quantification were maintained for 6 h, and for protein expression analysis for 24 h. For ROS assessment, cells were pretreated with ACN for 2 h prior to DOX exposure. All results are expressed as fold-change relative to OS control and presented as mean (*n* ≥ 3) ± SD. Different letters indicate statistically significant differences between treatments, as determined by ANOVA. Symbols (− and +) indicate absence or presence of DOX IC_50_, respectively. mRNA and protein expression levels were quantified as described in Section 2.

The responses of Nrf2 downstream genes expressed by AREs were also evaluated. The results showed that Hmox1 mRNA levels tended to be upregulated by OS without reaching significance. However, ACN treatments at 0.5 × IC_50_, 1 × IC_50_, and 1.5 × IC_50_ upregulated Hmox1 mRNA levels up to 1.92-, 5.33-, and 2.57-fold of OS-control, respectively (Figure 6a).

HO-1 is the protein encoded by the Hmox1 gene and it was upregulated by OS with ACN treatments enhancing such upregulation. The relative HO-1 band intensities in cells treated with ACN at 0.5 × IC_50_, 1 × IC_50_, and 1.5 × IC_50_ were 2.42-, 2.34-, and 1.34-fold of OS-control, respectively (Figure 6b). In cancer cells, ROS induce HO-1 as a defense mechanism. HO-1 helps cancer cells cope with OS, promoting survival, proliferation, and therapy resistance. Therefore, HO-1 is a potential therapeutic target, especially in cancers with high OS. Similarly, NQO1 is a double-edged sword in cancer because it protects cancer cells from ROS and promotes survival. But its overexpression may provide a therapeutic window to selectively generate ROS and kill cancer cells.

NQO1 mRNA levels did not increase by OS, but treatment with ACN at concentrations of 0.5 × IC_50_, 1 × IC_50_, and 1.5 × IC_50_) upregulated them up to 3.84-, 4.10-, and 3.86-fold of OS-control, respectively (Figure 6c). At protein levels, OS increased NQO1 expression compared to the DMSO control. ACN treatment at 0.5xIC_50_ maintained the OS-induced ROS levels; however, ACN treatments at higher doses prevented the OS-induced upregulation, maintaining NQO1 protein levels comparable to those observed under resistance-maintaining conditions (Figure 6d).

ROS levels aligned with changes in NQO1, being induced by OS, while low ACN dose (0.5 × IC_50_) maintained the OS-induced ROS levels. However, ACN at higher doses prevented ROS upregulation (Figure 6e).

This matches the findings in autophagy and ROS where impairment leads to sustained stress and a heightened inflammatory response due to the accumulation of ROS and damaged organelles, potentially surpassing the cellular inflammation and stress threshold and triggering cell death. It has been previously reported that ACN and DOX have synergistic effects in cancer cells, and this synergism might be explained through this mechanism [22].

### 3.4. ACN Modulates Antioxidant Enzyme Activity Associated with Drug Resistance

**Figure 7:**
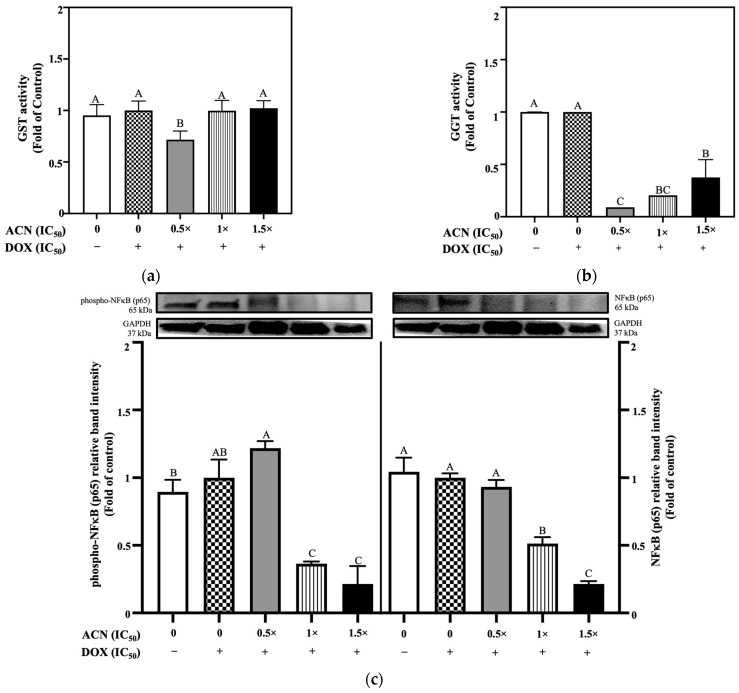
ACN modulates antioxidant enzyme activity and inflammatory signaling in 4T1-DR cells under oxidative stress. (**a**) GST activity in the presence of DOX IC_50_ following ACN treatment at different IC_50_ ratios. (**b**) GGT activity under the same conditions. (**c**) Protein expression levels of phosphorylated NF-κB p65 (**left**) and basal NF-κB (**right**) p65. For all treatments, 4T1-DR cells were incubated with ACN at various IC_50_ ratios in the presence of DOX at 10.75 µg/mL. Enzyme activities and protein levels were quantified as detailed in Section 2 and expressed as fold-change relative to control. Data are presented as mean (*n* ≥ 3) ± SD. Different letters indicate statistically significant differences between treatments, as determined by ANOVA. Symbols (− and +) indicate absence or presence of DOX IC_50_, respectively.

Overactivated GST and GGT enzymes have been reported in DR cells as a mechanism to lower drug’s toxicity and avoiding cell death. Their targeting is suggested as a therapeutic approach to resensitize cells to drugs’ effects [10]. Results showed that GST and GGT enzyme activities were not induced under OS, supporting the DR phenotype by not showing a trigger after OS exposure. GST enzymatic activity was significantly reduced following ACN administration only at 0.5 × IC_50_ down to 0.71-fold of OS-control, while higher ACN concentrations (1 × IC_50_, and 1.5× IC_50_) maintained GST activity like both controls (Figure 7a).

Furthermore, GGT activity was inhibited by all ACN doses, but with highest potency at 0.5 × IC_50_ (0.09-fold of OS-control) (Figure 7b). These results may be relevant considering that elevated GGT levels have been observed in various cancers (e.g., liver, pancreatic, breast) and associated with cancer progression, therapeutic resistance, and poor prognosis, specifically in breast cancer [7].

NF-κB protein levels showed a higher presence of phospho-NF-κB (left side) and no changes in basal NF-κB levels (right side) when treated with ACN 0.5× IC_50_ (Figure 7c), while higher ACN doses showed lower inflammation effects. Results from NF-κB protein expression align with changes in phospho-p62 and p62, with ROS and GST activity at ACN 0.5 × IC_50_ sustaining elevated OS-induced ROS, suppressing GST antioxidant activity, and impairing autophagy as key factors contributing to increased inflammation. The combination of ACN at 0.5 × IC_50_ and DOX 1 × IC_50_ seemed to be the optimal treatment to lower the activity of both GST and GGT enzymes, along with modulation of HO-1 and NQO1 protein levels at which sustained inflammation and ROS-induced damage may exceed the cells’ repair capacity and contribute to killing the DR BC cells. However, future studies in 4T1-DR cells are guaranteed to determine synergistic ACN-DOX combinations.

### 3.5. Doxorubicin Dose–Dependent Alterations in Oxidative Stress Pathway Responses

**Figure 8:**
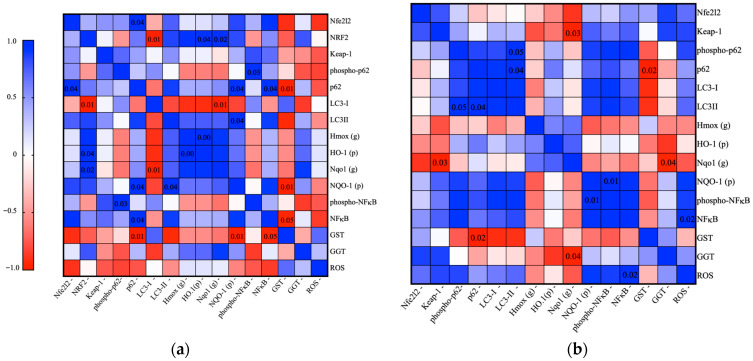
Pearson correlation heatmaps illustrate shifts in gene, protein, and antioxidant enzyme activity interrelationships in 4T1-DR cells under resistance-maintaining and OS conditions. (**a**) DOX resistance-maintaining dose and (**b**) DOX IC_50_ dose. Color intensity and hue indicate the strength and direction of correlation (blue: positive, red: negative, white: none), and numbers presented within the heatmap denote statistically significant correlations with *p*-values below 0.05. The figure demonstrates treatment-dependent changes in molecular mechanism, revealing distinct patterns of gene (g), protein (p), and biochemical activity interplay between the two conditions.

Spearman correlation heatmaps illustrate the relationships among genes, proteins, and activities in ACN-treated cells under two conditions: resistance-maintaining (exposed to DOX at a resistance-maintaining dose of 100 nM; Figure 8, left panel) and OS (DOX at the IC_50_ dose; Figure 8, right panel).

Overall, a shift in correlation patterns was observed between resistance-maintaining and OS conditions, likely to reflect cellular adaptation to OS through differential responses.

For resistance-maintaining conditions, strong correlations were observed among markers downstream of the Nrf2 pathway, including NRF2 at the protein level, the genes Nqo1 and Hmox1, and the HO-1 protein. At these conditions, both phosphorylated forms of p62 and NF-κB as well as their basal forms were correlated to each other, indicating a pattern of phosphorylation as a treatment response. Overall, the heatmap reflects a canonical response involving markers from the Nrf2 pathway under resistance-maintaining conditions.

Under OS, correlation patterns shifted. The autophagic markers p62 and LC3-I and LC3-II were more tightly correlated indicating a response in autophagy at this dose. AREs did not show any strong correlations with any antioxidant markers; however, NQO-1 protein was strongly correlated with NF-κB. Additionally, NF-κB exhibited a high correlation with ROS, suggesting an inflammation response by the presence of OS. No distinct correlation was detected in any ARE-activated genes/proteins, strengthening the theory of a shift in the Nrf2 antioxidant pathway under OS moving to an autophagic response.

**Figure 9:**
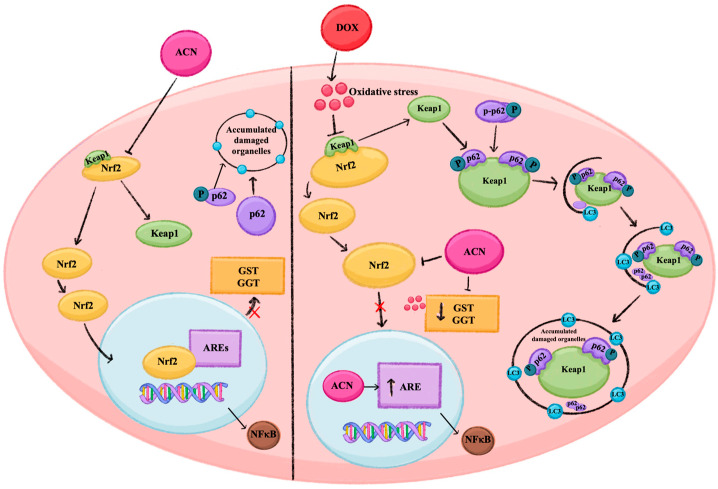
Summary of pathway response cell to ACN at 0.5 × IC_50_ in 4T1-DR cells under resistance-maintaining and oxidative stress conditions triggered by DOX. The figure illustrates treatment-dependent changes in the pathway response to ACN treatments, when under resistance-maintaining conditions (**left** side) or DOX-induced oxidative stress (**right** side). Solid arrow indicates activation, stimulation or promotion of the following marker. Blunt arrows indicate inhibition of the following marker. A blocked solid arrow indicates that promotion or activation is prevented from occurring. Abbreviations used in the figure: Nrf2—nuclear factor erythroid 2–related factor 2, Keap1—Kelch-like ECH-associated protein 1, p62—sequestrosome p62, LC3—microtubule-associated protein 1A/1B-light chain 3, ACN—anthocyanin-rich extract, DOX—doxorubicin, GST—glutathione S-transferase, GGT—gamma-glutamyltransferase, ARE—antioxidant response elements, NFκB—nuclear factor kappa-light-chain-enhancer of activated B cells.

In summary, the heatmap under resistance-maintaining conditions reveals tighter, more cohesive gene/protein regulation on the canonical pathway, while the OS heatmap shows an autophagic response and a pathway-specific dysregulation, reflecting biological shifts in response to OS. A summary of the effects of ACN in cells under resistance-maintaining and OS conditions at the lowest dose (0.5 × IC_50_) is illustrated in Figure 9.

## 4. Discussion

This study showed how ACN treatments impact molecular mechanisms linked to DR differently depending on resistance-maintaining or OS conditions. The effects of ACN in the antioxidant machinery in both the canonical and non-canonical pathway was explained. At resistance-maintaining conditions, Nrf2 was increased at both mRNA and protein levels, indicating possible Nrf2 nuclear translocation which was signaled by the expression of Nrf2-target genes whose transcription is promoted by AREs. No changes in Keap1 protein under these conditions suggest that Nrf2 activation may result from compounds in ACN upregulating this transcription factor, leading to its increased expression and accumulation. This promotes its translocation without affecting Keap1. These findings align with previous reports showing that polyphenols can directly induce Nrf2, causing its nuclear translocation and thereby activating the antioxidant defense machinery [34]. When this mechanism is activated, cells can sustain OS induced by chemotherapeutic drugs, which is a major contributor to DR [3]. It has been reported that Nrf2 activation in cancer-initiated cells exhibits a dual role: while it can protect against oxidative damage, it may also act as a pro-tumorigenic factor by driving mutations and signaling pathways that promote cellular proliferation and survival. Furthermore, elevated Nrf2 activity in tumors is associated with poor prognosis and recurrence, highlighting that therapeutic strategies to activate or inhibit Nrf2 must be carefully tailored to the specific disease [35]. Therefore, its inhibition has been proposed as a therapeutic approach to increase cellular susceptibility to oxidative damage, thereby sensitizing cells to DOX [36].

The activation of the Nrf2–Keap1 pathway by ACN under resistance-maintaining conditions appears to influence autophagic processes. At higher ACN doses, reduced phospho-p62 suggests limited clearance of Keap1 through autophagy, indicating impairment across all concentrations. Interestingly, this impairment seemed more pronounced at lower doses, which was accompanied by changes in LC3 dynamics, further pointing to autophagy activation as a stress-responsive mechanism that preserves cellular homeostasis. The concurrent engagement of Nrf2 signaling and autophagy supports the degradation of damaged organelles, promoting cell survival. In DR cancer cells, elevated autophagic activity serves as an adaptive advantage, enabling the removal of harmful compounds and organelle damage, thereby sustaining survival even under chemotherapeutic pressure [6].

Nevertheless, it has been reported that inducing autophagy and blocking its flux leads to the accumulation of damaged organelles and ultimately induces cell death. A study using the polyphenol nobiletin in the tamoxifen-resistant human ovarian cancer cell line SKOV3 evaluated its potential to suppress cell growth. The findings demonstrated that nobiletin impaired autophagic flux, as evidenced by increased expression of LC3-II and p62 proteins. The authors concluded that suppression of autophagic flux was directly linked to cancer cell death through the induction of apoptosis [37]. These results align with the findings presented here, which suggest that although autophagy is initiated, ACN impairs the process at the lowest dose, as indicated by the sustained presence of both phospho-p62 and p62. Notably, a separate study investigating the effects of cyanidin on renal cell carcinoma 786-O and ACHN cells revealed that cyanidin downregulated autophagy-related (ATG) proteins, which are essential for maintaining autophagic flux following autophagosome formation. This supports the proposed mechanism by which ACN disrupts autophagic flux in the current study [38]. The role of polyphenols in modulating autophagy is well-documented, with several studies reporting that they can impact autophagy at different levels, including at the late-stage by impairing autophagic flux, thereby promoting cell death by inducing cellular stress through the accumulation of damaged organelles [39].

Induction of the Nrf2–Keap1 pathway under resistance-maintaining conditions was accompanied by increased expression of NQO1 and HO-1 at both gene and protein levels at higher ACN doses which was contrasted by ACN at low dose with no significant induction of these markers, except for NQO1 protein. This suggests that ACN fails to activate the antioxidant pathway. As a result, its antioxidant function is insufficient to eliminate the drug and mitigate its effects on cell viability. This was further supported by the lack of change in GGT enzyme activity at this dose, while GST activity was suppressed across all ACN treatments. A study reported that a fraction rich in flavonoids and phenolic compounds extracted from Tamarindus indica seed extract exhibited the strongest GST inhibitory effect in the human BC MCF-7 cell line, an effect linked to its potent anticancer effects in this model [40]. These findings suggest that ACN may act as direct inhibitors of GST enzyme activity in 4T1-DR BC cells, a mechanism also reported for various phytochemicals showing therapeutic potential to inhibit cancer cells [41]. Both GST and GGT enzymes are related to detoxification of chemotherapy and their enhanced activity has been related to the reduction in ROS, therefore maintaining cellular homeostasis and preventing apoptosis [7].

No change in ROS was observed at any ACN treatment compared to the control under resistance-maintaining conditions. This result is consistent with prior research showing that activation of the Nrf2-Keap1 antioxidant pathway sustains redox homeostasis without triggering cell death. Such stability reflects adaptive redox reprogramming, wherein polyphenols help regulate OS without necessarily inducing changes in overall ROS levels [34]. The absence of ROS induction alongside changes in inflammation with upregulation of the *p*-NF-κB/NF-κB (p65) ratio by ACN treatment at 0.5x IC_50_, followed by downregulation at higher ACN doses, suggests that ACN may exert a pro-oxidant effect through suppression of GST enzyme activity while GGT is not induced. This indicates that only a low dose of ACN may sensitize the cells to DOX treatment since higher doses of ACN induced antioxidant response and reduced inflammation, thus promoting homeostasis and helping cells to evade cell death by OS.

Notably, ACN treatments produced distinct outcomes depending on dose and either OS or resistance-maintaining conditions, underscoring the potential of polyphenols as adjuvants in chemotherapy. While Nrf2 is commonly degraded via the Keap1-dependent pathway, studies have shown that it can also be degraded by PI3K-dependent mechanisms or epigenetic modifications, including DNA methylation, histone alterations, and post-translational changes [7,35,42]. Polyphenols can act as Nrf2 activators or inhibitors. As activators, polyphenols may induce Nrf2 directly or modify specific cysteine residues on Keap1, thus preventing binding and targeting for Nrf2 degradation. As inhibitors, polyphenols can downregulate PI3K-Akt and ERK signaling pathways or cause epigenetic alterations that downregulate Nrf2. Through these mechanisms, polyphenols may sensitize cancer cells to therapy by inhibiting rather than activating Nrf2 [42,43,44]. This supports the observed Nrf2 degradation under OS in the present study.

While Keap1 downregulation typically reduces Nrf2 ubiquitination and enhances its activation, the absence of Nrf2 expression coupled with Keap1 downregulation strengthens the theory of direct downregulation of Nrf2 by ACN, rather than by the canonical pathway. Supporting this, a study investigating the effects of epigallocatechin gallate (EGCG), the predominant polyphenol in green tea, on tamoxifen-resistant MCF-7 BC cells found that EGCG treatment downregulated Nrf2 protein levels. This suppression occurred independently of Keap1, as its expression remained unchanged [45]. In a separate study, the flavonoid luteolin was tested in MCF-7 (BC), and Caco-2 (colon cancer) cell lines to evaluate its role in sensitizing cancer cells to therapy through Nrf2 inhibition. Luteolin treatment resulted in a dose-dependent decrease in Nrf2 expression. To determine whether this effect was Keap1-dependent, Keap1 was silenced, and the authors observed a similar downregulation of Nrf2 markers, confirming that luteolin can suppress Nrf2 independently of Keap1. These findings support the concept that certain polyphenols can act as inhibitors of Nrf2, bypassing canonical Keap1-mediated regulation [46]. The specific molecular mechanism in which ACN modulated the Nrf2 pathway is yet to be elucidated.

The downregulation of Keap1 observed in this study aligns with the activation of the non-canonical antioxidant regulatory pathway mediated by autophagy. Under high OS, cells may cause autophagic cell death by accumulating autophagosomes due to impaired autophagic flux [12]. Under these conditions, cells often activate the unfolded protein response (UPR), which in turn triggers autophagy as a protective mechanism [13]. This process involves modulation of key signaling molecules such as Akt, mTORC1, and AMPK [47], all of which have been reported to be influenced by ACN. In this study, the observed turnover from LC3-I to LC3-II strongly suggests enhanced autophagic activity at higher doses but impaired autophagy at the lowest ACN dose, similar to the results at resistance-maintaining conditions. ACN have previously been associated with downregulation of mTOR [24], a central negative regulator of autophagy. This supports the hypothesis that ACNs may promote autophagy through mTOR inhibition [48].

A study using the polyphenol resveratrol in the human non-small-cell lung cancer cell line A549 found that this compound can induce autophagy, as evidenced by the upregulation of LC3-II/LC3-I at the protein level and the downregulation of p62. These findings support the idea that polyphenols enhance sensitivity to chemotherapeutic agents through autophagy-mediated mechanisms, ultimately promoting cell death, a phenomenon previously described in the literature [44,49]. In a previous study, we demonstrated that ACNs can exert either antagonistic or synergistic effects when combined with DOX, depending on the dose [22]. In this study, the combination of ACN and DOX exhibited synergistic cytotoxic effects on breast cancer cells by enhancing OS and reducing the degradation of damaged organelles through autophagy, thereby triggering stress responses that lead to cell death. It has been reported that autophagic degradation products are transported to support cellular metabolism and repair mechanisms; thus, downregulation of autophagic flux also disrupts these essential processes aiding in cell death signaling [39].

Since Nrf2 was not present, no activation of AREs was expected. Nonetheless, an increase in NQO1 and HO-1 protein levels was detected in the OS control at protein levels. It has been reported that these enzymes can be upregulated in response to endoplasmic reticulum stress induced by DOX, thus explaining their upregulation in both the OS control and ACN treatments. Treatments with ACN increased HO-1 levels compared to the OS control at both mRNA and protein levels. The effect of certain polyphenols in NF-κB and its relation to HO-1 has been already discussed [50]. HO-1 is a known target gene of NF-κB, as its promoter contains binding sites for this transcription factor, suggesting a regulatory relationship between NF-κB activity and HO-1 expression [51]. In the present study, NF-κB was downregulated at the highest ACN concentration which also showed a downregulation in HO-1 levels, while they were both upregulated at the lowest ACN dose, demonstrating this correlation.

NQO1 was significantly upregulated by ACN at a lower dose, but its expression returned to levels comparable to the control group at higher ACN doses. Increased NQO1 expression is commonly associated with the cellular response to DNA damage, given its role in detoxifying reactive quinones and protecting against oxidative injury. Additionally, NQO1 is recognized as a target gene of NF-κB [52]. These results imply a correlation and potential mechanistic interplay between the responses of HO-1, NQO1, and NF-κB during cellular adaptation to oxidative stress.

The effect of ACN inhibiting both GST and GGT at the lowest dose can be compared to other polyphenol–drug combinations in previous studies. For instance, a study using MDA-MB-231 BC cells found similar results using a DOX–quercetin combination: DOX alone had no effect on GST activity, but the combination significantly reduced GST activity [53]. Likewise, a study evaluating the synergistic interaction of phytochemical fucoxanthin and DOX found the inhibition in GST activity in DR hepatocellular carcinoma (HepG-2) cancer cells [54]. It has been reported that GSH products generated by GST activity are often exported via multi-drug resistance transporters [7], which were previously reported to be downregulated by ACN [22], suggesting a coordinated action between both GST enzymes and drug metabolizing enzymes. This enzyme’s downregulation at the lowest ACN dose may reflect the dose-dependent nature of ACN-DOX interactions, which have been reported to be either antagonistic or synergistic [22].

Since GSH deficiency is known to trigger ferroptosis, a form of oxidative cell death driven by compromised GSH-dependent antioxidant defenses [55], and reduced GSH levels have been linked to increased sensitivity to chemotherapeutic agents [44], GGT suppression may contribute to ferroptosis activation and enhance DOX efficacy. Additionally, inhibition of Nrf2 further supports this mechanism by weakening the antioxidant response and promoting autophagosome–lysosome fusion, thereby disrupting cellular metabolism and repair processes that rely on autophagic degradation products and reinforcing ferroptosis induction [55].

Notably, ACN at 0.5 × IC_50_ maintained OS-induced ROS levels, a similar trend to HO-1, NQO1, and NF-κB. A previous study reported that curcumin-induced autophagy in the human HCT116 colon cancer cell line led to an increase in ROS levels. When co-treated with the antioxidant compound *N*-acetylcysteine (NAC), both ROS and autophagy levels were reduced, suggesting that autophagy in this context was correlated to ROS generation [56]. Another study using the human HT-29 colon cancer cell line showed that resveratrol alone increased autophagy and ROS levels, but the quenching of ROS led to reduced autophagy and autophagic cell death [57]. The current study revealed a similar pattern under DOX-induced OS, in which ACN at 0.5× IC_50_ maintained constant ROS levels while reduced antioxidant activities contributed to the cells unsustainable state of OS along impaired autophagy, leading to an increase in stress signaling which may push a tumor cell beyond breaking point regarding DNA damage and protein oxidation which leads to cell death.

## 5. Conclusions

The effect of the anthocyanin-rich fraction (ACN) extracted from DSC on DR TNBC 4T1 cells is highly context-dependent, varying according to resistance-maintaining conditions or DOX-induced OS. Under resistance-maintaining conditions, ACN did not stimulate a complete antioxidant response but rather disrupted autophagic flux and increased inflammatory signaling, ultimately initiating cellular stress responses that promote cell death.

A similar pattern of autophagy impairment was observed at the lowest ACN dose under conditions of elevated OS induced by higher DOX concentrations. In addition, under these conditions, ACN shifted its role in the antioxidant pathway, acting as a chemosensitizing co-adjuvant. In this capacity, it disrupted antioxidant defenses, impairing the detoxification of pro-oxidant drugs and enhancing cytotoxicity. Notably, ACN at its lowest dose suppressed both Nrf2 and Keap1 markers, indicating a lack of canonical pathway activation. This was further supported by reduced enzymatic activities of GST and GGT, leading to decreased GSH accumulation and limiting the enzymatic detoxification of DOX.

This mechanism appears to be linked to autophagy-mediated cell death, potentially through ferroptosis or apoptosis. The inhibition of Nrf2 not only weakens the antioxidant response but also promotes autophagosome–lysosome fusion, disrupting cellular metabolism and repair processes that depend on autophagic degradation products. ACN demonstrated the ability to enhance stress signaling and promote cell death under both resistance-maintaining and OS conditions. However, under OS, disruption of the Nrf2 pathway may lead to increased drug retention and elevated OS, further promoting cell death. To clarify the specific pathway involved, future studies should include assessments of gene knock-out and apoptosis-specific markers.

Overall, these findings highlight the dual role of ACN in modulating antioxidant mechanisms. Depending on the cellular context and dose treatment, ACN can either support or counteract DR, underscoring its potential as a context-specific therapeutic modulator. This study is limited to using a single cell line derived from a murine model. While this model provides valuable mechanistic insights, it may exhibit signaling pathway differences specific to this cell line. Although this study employed a murine TNBC cell line, the findings offer a promising perspective. This is supported by in vivo evidence demonstrating the safety and efficacy of anthocyanin-rich cherry phenolics alone and in combination with doxorubicin [18,21], highlighting the potential of phytochemicals as adjuvant therapies for other breast cancer subtypes. However, the applicability of these findings to human biology remains uncertain. Therefore, further validation through comparative studies using human TNBC cell lines is necessary to confirm these results.

Future perspectives for this research should also include validation of the current model using cells with varying resistance profiles to confirm generalization across different resistance stages, evaluation of dose- and context-dependent effects of anthocyanins under varying redox and metabolic conditions, investigation of potential interactions between cherry polyphenols and standard chemotherapeutic regimens beyond doxorubicin, and the exploration of biomarkers of response related to redox signaling and antioxidant pathways.

## Figures and Tables

**Table 1:** Primers used for gene expression analysis and their sequences.

Primer	Sequence
Nfe2l2	Forward 5′-CATTCCCGAATTACAGTGTC-3′
	Reverse 5′-GGAGATCGATGAGTAAAAATGG-3′
Hmox1	Forward 5′-CATGAAGAACTTTCAGAAGGG-3′
	Reverse 5′-TAGATATGGTACAAGGAAGCC-3′
Nqo1	Forward 5′-CCTTTCCAGAATAAGAAGACC-3′
	Reverse 5′-AATGCTGTAAACCAGTTGAG-3′
Rpl-19	Forward 5′-GAAGGTCAAAGGGAATGTGTTCA-3′
	Reverse 5′-CCTTGTCTGCCTTCAGCTTGT-3′

**Table 2:** Comparative cell viability of parental 4T1 cells and drug-resistant 4T1-DR cells following treatment with varying concentrations of doxorubicin (DOX) *.

	4T1 Parental	4T1-DR
DOX (µg/mL)	Cell Viability (% of Control) (Mean ± SD)	Cell Viability (% of Control) (Mean ± SD)
0	100 ± 8.34	100 ± 5.32
2.5	24.43 ± 2.36	79.43 ± 4.90
5	13.17 ± 0.96	67.31 ± 3.26
10	10.49 ± 0.94	51.88 ± 2.47
20	9.38 ± 1.21	38.35 ± 2.59

* Cell viability is expressed as percentage of control. Data shows mean (*n* ≥ 3) independent treatments ± SD).

**Table 3:** Comparative cell viability of parental 4T1 cells and drug-resistant 4T1-DR cells following treatment with various concentrations of anthocyanin-rich extract (ACN) *.

	4T1 Parental	4T1-DR
ACN (µg C3R Eq./mL)	Cell Viability (% of Control) (Mean ± SD)	Cell Viability (% of Control) (Mean ± SD)
0	100 ± 3.8	100 ± 3.5
25	71 ± 5.6	81 ± 3.5
50	39 ± 2.9	77 ± 2.7
100	9 ± 0.5	24 ± 1.9

* Cell viability is expressed as percentage of control. Data shows mean (*n* ≥ 3) independent treatments ± SD).

**Table 4:** Comparative cell viability of drug-resistant 4T1-DR cells following treatment with anthocyanin-rich extract (ACN) and pure cyanidin-3-O-rutinoside (C3R) standard at varying concentrations *.

	ACN	C3R
ACN (µg C3R Eq./mL)	Cell Viability (% of Control) (Mean ± SD)	Cell Viability (% of Control) (Mean ± SD)
0	100 ± 6.3	100 ± 6.3
10	95 ± 4.6	97 ± 4.5
20	88 ± 6.0	98 ± 4.2
40	69 ± 6.1	95 ± 5.0
60	47 ± 4.9	88 ± 8.6

* Cell viability is expressed as percentage of control. Data shows mean (*n* ≥ 3) independent treatments ± SD).

**Table 5:** Comparative cell viability of drug-resistant 4T1-DR cells following treatment with anthocyanin-rich extract (ACN) alone and in combination with doxorubicin (DOX) at a resistance-maintaining dose (100 nM) *.

	ACN	ACN + DOX
ACN (µg C3R Eq./mL)	Cell Viability (% of Control) (Mean ± SD)	Cell Viability (% of Control) (Mean ± SD)
0	100 ± 0.82	98.94 ± 3.3
50	92.77 ± 1.32	104.11 ± 1.62
100	62.42 ± 3.73	60.38 ± 4.07
150	16.12 ± 0.85	15.22 ± 0.71

* Cell viability is expressed as percentage of control. Data shows mean (*n* ≥ 3) independent treatments ± SD).

## Data Availability

The raw data supporting the conclusions of this article will be made available by the authors upon request.

## Abbreviations

The following abbreviations are used in this manuscript:

ACN	Anthocyanin-rich fraction
ARE	Antioxidant response element
BC	Breast cancer
BSA	Bovine Serum Albumin
C3R	Cyanidin 3-O-Rutinoside
CDNB	1-Chloro-2,4-dinitrobenzene
DOX	Doxorubicin
DR	Drug resistance
DSC	Dark sweet cherries
FBS	Fetal bovine serum
GGT	γ-glutamyl transferase
GSH	Glutathione
GST	Glutathione S-transferase
H2DCFDA	2,7-Dichlorofluerescin diacetate
HO-1	Heme oxygenase 1
Keap1	Kelch-like ECH-associated protein 1
NQO1	NADPH quinone oxidoreductase 1
Nrf2	Nuclear factor erythroid 2–related factor 2
OS	Oxidative stress
P/S	Penicillin–streptomycin antibiotic mix
ROS	Reactive oxygen species
TNBC	Triple negative breast cancer
